# Sacral neuromodulation improves functional outcome and quality of life in patients with pouch dysfunction after pan proctocolectomy for ulcerative colitis

**DOI:** 10.1007/s10151-025-03140-4

**Published:** 2025-05-03

**Authors:** F. Drissi, M. Di Giuseppe, C. Korkmaz, A. Bourreille, X. Tréton, F. Ris, G. Meurette

**Affiliations:** 1 Chirurgie Cancérologique Digestive et Endocrinienne, Institut des Maladies de l’Appareil Digestif, Hôtel Dieu, University Hospital of Nantes, 1 Place Alexis Ricordeau, 44093 Nantes Cedex 01, France; 2 Department of Surgery, Ospedale Regionale di Bellinzona e Valli, via Ospedale 12, 6500 Bellinzona, Switzerland; 3 Hépato-Gastro-Entérologie, Institut des Maladies de l’Appareil Digestif, Hôtel Dieu, University Hospital of Nantes, 1 Place Alexis Ricordeau, 44093 Nantes Cedex 01, France; 4 Institut des MICI, Groupe Hospitalier Privé Ambroise-Paré-Hartmann, Neuilly, France; 5 Division of Digestive Surgery, University Hospital of Geneva, Geneva, Switzerland; 6 Service de Chirurgie Digestive, Clinique Jules Verne, 2-4 route de Paris, 44314 Nantes Cedex 3, France

**Keywords:** Sacral neuromodulation, Ileal pouch-anal anastomosis, Ulcerative colitis, Fecal incontinence, Quality of life

## Abstract

**Background:**

Postoperative frequency of bowel movements and impaired fecal continence (FI) has a negative impact on quality of life following ileal pouch-anal anastomosis. Sacral neuromodulation (SNM) is a validated treatment of FI, but its effectiveness in patients with ileal pouch-anal anastomosis (IPAA) has been poorly reported. The aim was to assess the results of SNM in patients with IPAA suffering from functional disorders and to compare these results with those of patients routinely treated by SNM for FI.

**Methods:**

A 3-week test phase was performed before definitive implantation of the pulse generator. Patients’ data were prospectively gathered in a dedicated registry. Patients with IPAA were then compared with a matched-paired control group of patients routinely treated by SNM for FI.

**Results:**

Between 2007 and 2020, 14 patients with IPAA were tested and 12 (85%) were implanted. This group was compared with a matched group of 20 patients implanted for FI. After a mean follow-up of 4.8 [0.5–16] years, there was a significant decrease of weekly leaks (29 versus 2; *p* = 0.01), decrease in Wexner score (15 versus 10.8; *p* = 0.01), and an improvement in quality of life (fecal incontinence quality of life (FIQOL) 1.853 versus 2.42; *p* = 0.01). Patients with IPAA evolved equally as compared with the control group in terms of Wexner score and quality of life at 6 months, 1 year, and 2 years.

**Conclusions:**

SNM provides a significant decrease of leaks and improves Wexner scores and quality of life in patients with IPAA. The effectiveness seems comparable to patients routinely treated by SNM for FI. SNM indications could be extended to patients with IPAA who present with bad functional outcome.

## Introduction

Panproctocolectomy (PPC) and reconstructive ileal pouch-anal anastomosis (IPAA) are the gold standard procedures in patients with refractory ulcerative colitis (UC) or with familial adenomatous polyposis (FAP). While almost all patients with FAP should undergo PPC-IPAA, it is estimated that about 10–20% of patients with UC will be concerned over time [1]. Although PPC-IPAA is deemed to solve the disease, functional outcome can be impaired by pouchitis, increased stool frequency, fecal urgencies, and fecal incontinence. The mean reported stool frequency ranges between 6–7/day, with 1–2 stools by night, and approximately 36–57% of the patients will use antidiarrheal medications [2]. As surgery implies colorectal resection, despite sphincter preservation, fecal incontinence can be as high as 18% at day time and 29% at night [3]. As a consequence, quality of life is negatively impaired [4–6].

In those patients with fecal incontinence following IPAA, after having ruled out a complication (anastomotic stricture, fistula, chronic pelvic sepsis, or pouchitis), treatment options are generally limited to lifestyle modifications, antidiarrheal medications, and anoperineal retraining. Recently, sacral neuromodulation (SNM) has been proposed in this context before discussing ileostomy [7, 8].

SNM is a validated treatment for fecal incontinence, providing significant improvement in 80% of the patients for whom the test phase is successful [9]. The procedure involves the implantation of a tined lead near the S3 sacral nerve root, which applies a permanent low-voltage electrical stimulation through a pulse generator. SNM provides a decrease of leakage episodes, an increase of deferment time, and a correlative improvement of quality of life for patients with fecal incontinence [10]. Although the mechanisms of action of SNM are still partially understood, it is acknowledged that the effects not only include an improvement in sphincter function but also a regularization of colonic motricity and rectal emptying. On the basis of animal studies, it has been demonstrated that SNM effects are mediated by an afferent spinal and efferent vagal pathway, passing through the nucleus of the solitary tract located in the medulla oblongata [11]. This explains that SNM is likely to exert not only local (anal sphincter) but also central effects on bowel function.

Currently, the results of SNM in improving functional outcome following IPAA have been poorly reported in literature. A meta-analysis and a retrospective study reported the results of SNM in patients with IPAA with fecal incontinence. All studies are based on small samples; however, an improvement of fecal incontinence and a decrease of stool frequency has been reported. Despite these promising results, SNM is still poorly offered to patients with IPAA who have bad functional outcome and is not a validated indication as such.

The aim of this study was to assess the results of SNM in patients with poor functional outcome following IPAA and to compare these results with those of patients routinely treated by SNM for fecal incontinence.

## Methods

### Patients and data

Between November 2007 and March 2020, all the patients undergoing SNM for fecal incontinence following IPAA for UC in the University Hospital of Nantes were retrospectively identified from a prospectively maintained registered database (CNIL no. 1745462v0). Data collected were: patient characteristics (age, gender, body mass index, and surgical history), fecal incontinence characteristics (type of fecal incontinence, duration, and primary cause), sacral neuromodulation procedure (sacral nerve root/side), and outcome (adverse events, bowel diary, Wexner score, and FIQOL score). Patients with minimal follow-up data ≥ 1 year were included in the analysis. 

As of now, SNM is not a common and validated approach in the treatment of fecal incontinence in patients with IPAA; therefore, we compared the results of patients with IPAA with a matched group of patients treated for fecal incontinence as part of usual practice who were included in the SNM registry (2002–2020). Matching process was blinded to the study outcomes and performed according to age, gender, and preoperative incontinence severity (Wexner score).

### Sacral neuromodulation procedure

All patients underwent a 3-week test period after tined lead positioning. If an improvement of ≥ 50% of functional disorders was achieved, a permanent Interstim^™^ neurostimulator system (Medtronic, Minneapolis, MN, USA) was implanted. Antibiotic prophylaxis was delivered during the procedure in accordance with current recommendations. Follow-up visits were scheduled at 6 months and annually. Postoperative complications and functional outcomes were controlled at this time.

### Statistical analyses

Statistical analyses were performed using R (R Core Team, 2021). R: A language and environment for statistical computing. R Foundation for Statistical Computing, Vienna, Austria. Quantitative data were expressed as mean with corresponding standard deviation and extreme values or median. Categorical values were expressed as *n* (%). Comparison of qualitative and quantitative data was performed using a Fischer’s exact test and the Student’s *t* test, respectively. A paired samples *t*-test was used to compare pre- and post-observations in same subjects. Comparisons were considered significant at a *p* value < 0.05.

## Results

### Patients

Between November 2007 and March 2020, 14 patients underwent a SNM test for fecal incontinence following IPAA for UC. The mean age of the patients was 52 ± 11.1 years and eight patients were males. Two (14.3%) patients had an American Society of Anesthesiologists (ASA) score ≥ 3. No obstetrical anal sphincter injuries were reported among female patients. Pretreatment endoanal ultrasound was performed in four out of six female patients and did not find any sphincter disruption. Anorectal manometry was performed in three patients: two patients had low resting and squeeze anal pressure and two patients had dyssynergia. 

The mean time interval between IPAA and SNM procedures was 5.1 ± 3.4 years. The mean follow-up duration was 4.8 ± 4.3 years. No adverse events were reported following test period or definitive implantation.

At baseline, the mean stool frequency was 8.9 ± 2.8/day. Patients experienced a mean of 29.1 ± 28.5 leaks per week. The mean Wexner score was 14.8 ± 3.

### Functional outcomes

**Fig. 1:**
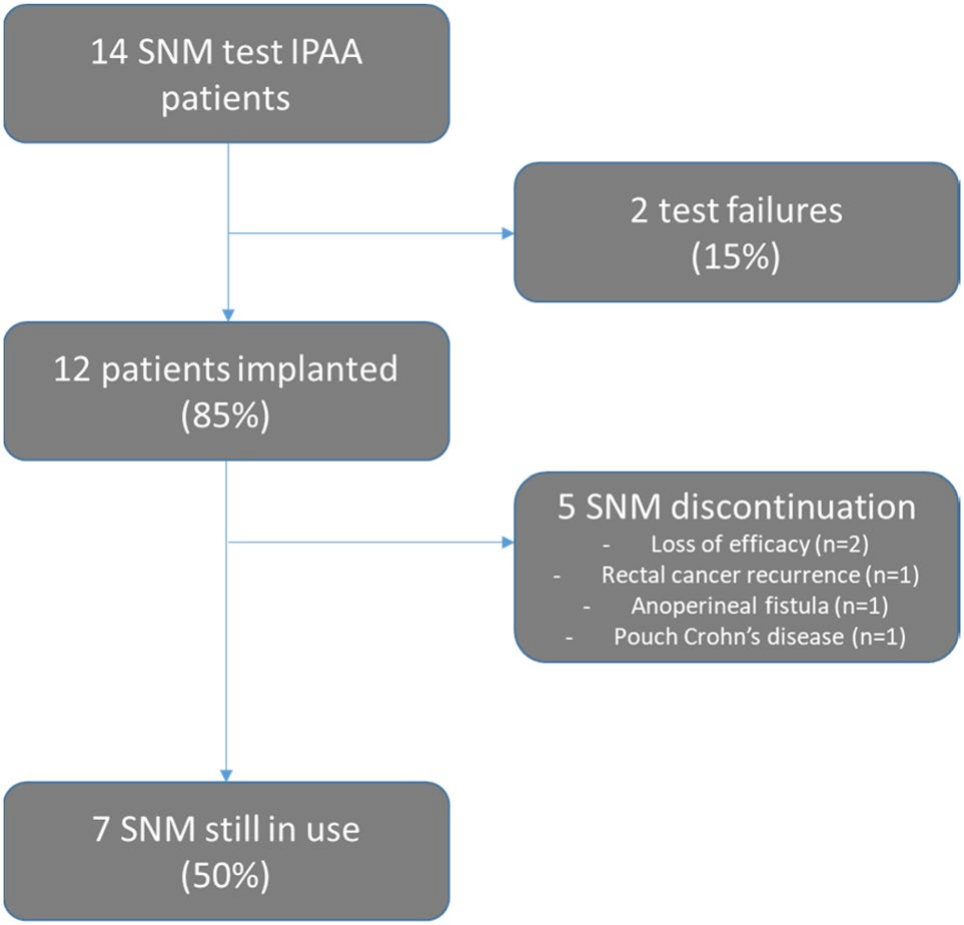
Study flowchart

A total of 12 (85.7%) patients were offered definitive pacemaker implantation after a successful test (Fig. 1). Among the two patients who failed the SNM test, one was given definitive ileostomy 2 years after. SNM therapy was discontinued, at a mean time of 34 ± 41.5 months, in five (35.7%) patients owing to loss of efficacy (*n* = 2), local recurrence of rectal cancer (*n* = 1), complex anoperineal fistula (*n* = 1), and anastomotic stricture due to Crohn’s disease of the pouch (*n* = 1). In these patients, pouch explantation and definitive ileostomy was performed. SNM was still in use in seven (50%) patients at the end of the follow-up.

After a mean follow-up of 4.8 years, there was a significant decrease of stool frequency (8.9–6.1, *p* = 0.004), number of leaks per week (29.1–2, *p* = 0.01), and Wexner incontinence score (15–10.8, *p* = 0.001) and a significant improvement in quality of life (FIQOL score 1.85–2.42, *p* = 0.01).

**Fig. 2:**
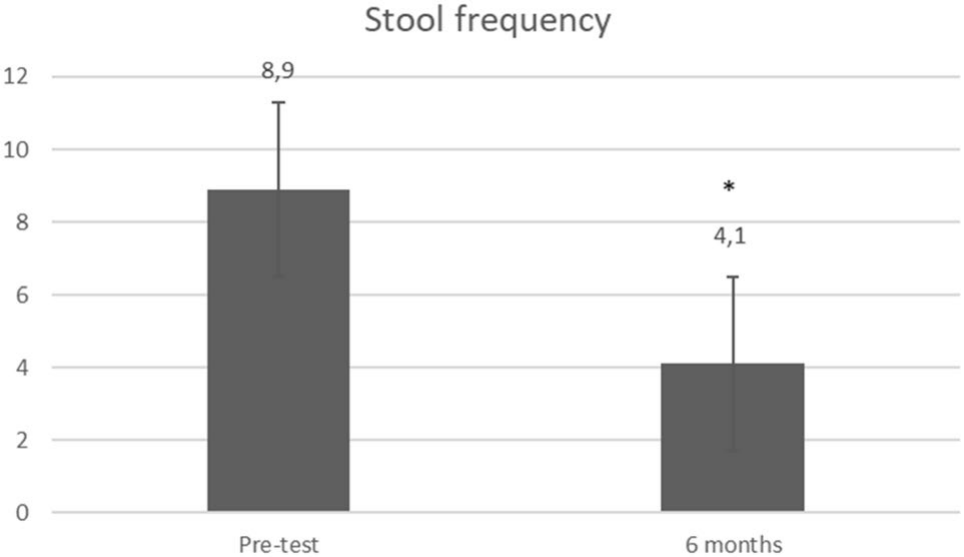
Stool frequency. Stool frequency is expressed by number of stools per day at baseline (*n* = 12) and at 6 months (*n* = 8); *Indicates a significant difference as compared with baseline value

**Fig. 3:**
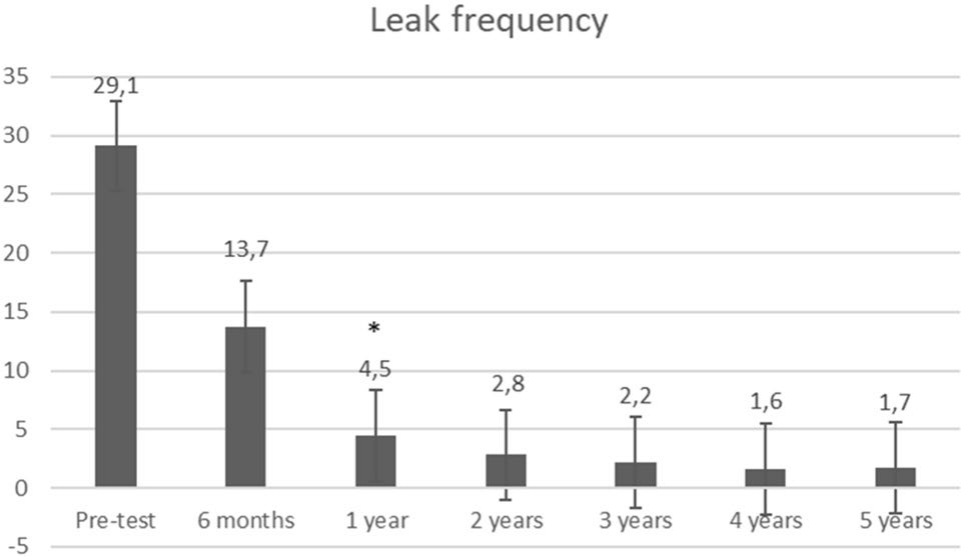
Leak frequency. Leak frequency is expressed by number of leaks per week at baseline (*n* = 12), 6 months (*n* = 8), 1 year (*n* = 9), 2 years (*n* = 6), 3 years (*n* = 5), 4 years (*n* = 4), and 5 years (*n* = 4); *Indicates a significant difference as compared with baseline value

**Fig. 4:**
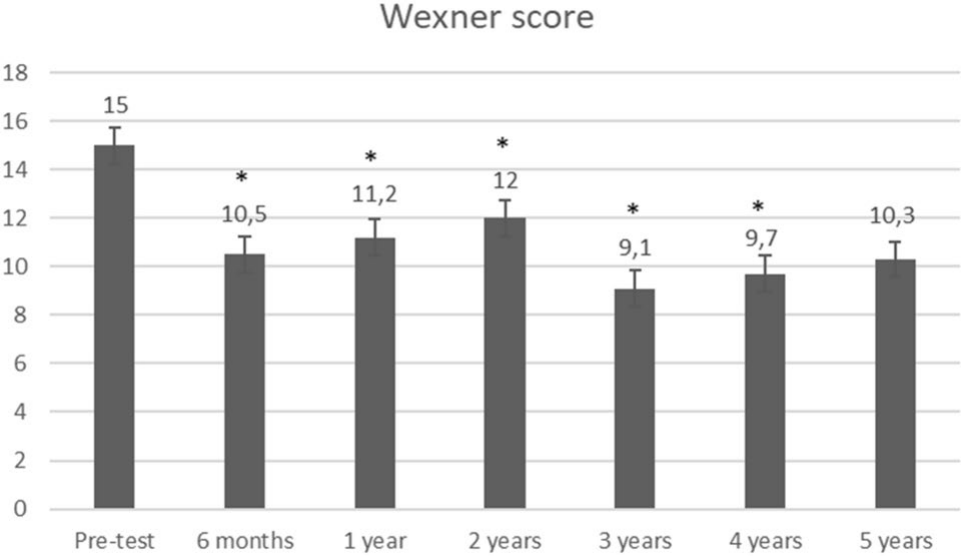
Fecal incontinence severity. Fecal incontinence severity is expressed by mean Wexner scores at baseline (*n* = 12), 6 months (*n* = 11), 1 year (*n* = 10), 2 years (*n* = 9), 3 years (*n* = 7), 4 years (*n* = 6), and 5 years (*n* = 3); *Indicates a significant difference as compared with baseline value

Stool frequency significantly decreased from 8.9 to 4.3 (*p* = 0.0001) at 6 months (Fig. 2). Further systematic data collection regarding number of stools was missing. Over time, the number of leaks per week dramatically decreased from 29.1 to 13.7 (*p* = 0.05), 4.5 (*p* = 0.01), 2.8 (*p* = 0.18), 2.2 (*p* = 0.28), 1.6 (*p* = 0.14), and 1.7 (*p* = 0.21), respectively, at 6 months, 1 year, 2 years, 3 years, 4 years, and 5 years (Fig. 3). The mean Wexner score decreased from 15 to 10.5 (*p* = 0.0005), 11.2 (*p* = 0.002), 12 (*p* = 0.01), 9.1 (*p* = 0.003), 9.7 (*p* = 0.003), and 10.3 (*p* = 0.059), respectively, at 6 months, 1 year, 2 years, 3 years, 4 years, and 5 years. There was an improvement in quality of life as FIQOL scores increased from 1.85 to 2.58 (*p* = 0.0001), 2.56 (*p* = 0.003), 2.32 (*p* = 0.03), 2.81 (*p* = 0.02), 2.63 (*p* = 0.02), and 2.40 (*p* = 0.12), respectively, at 6 months, 1 year, 2 years, 3 years, 4 years, and 5 years (Fig. 4).

**Fig. 5:**
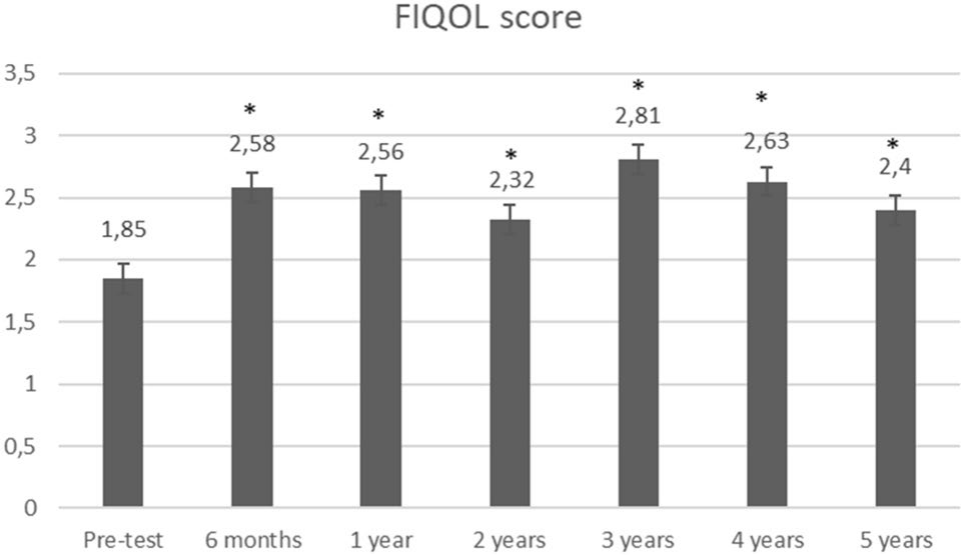
Quality of life. Quality of life is expressed by FIQOL scores at baseline (*n* = 12), 6 months (*n* = 10), 1 year (*n* = 9), 2 years (*n* = 8), 3 years (*n* = 6), 4 years (*n* = 6), and 5 years (*n* = 3); *Indicates a significant difference as compared with baseline value

Gender and interval time between IPAA and SNM implantation did not influence functional outcomes except for a better FIQOL score in patients receiving late implantation, more than 3 years after IPAA formation (FIQOL 2.22 early implantation versus 2.99 late implantation, *p* = 0.04) (Fig. 5).

### Comparison of patients with IPAA and those with fecal incontinence as a control group

The control group consisted of 20 patients, with mean ages of 52.6 ± 10.6 years, routinely treated by SNM for fecal incontinence due to sphincter lesion (*n* = 8), idiopathic fecal incontinence (*n* = 5), colorectal surgery sequelae (*n* = 5), or pelvic radiotherapy (*n* = 2) (Table 1). The two groups did not differ regarding matching criteria (age, gender, and pre-test Wexner score).

**Table 1:** Patient characteristics of control group

Patient characteristics (*n* = 20)	
Age (years)	52.6 ± 10.6 [20–75]
Gender	
Male	12 (60)
Female	8 (40)
Body mass index (kg/m^2^)	25.1 ± 3.5 [18–34]
Follow-up (years)	6.9 ± 2.6 [1.6–11.6]

Continuous data are expressed by mean value, standard deviation, and range. Categorical variables are summarized by the number of patients in each category and the corresponding percentages.

**Fig. 6:**
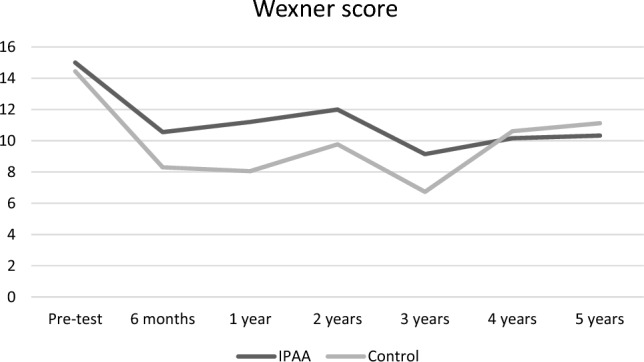
Comparison of fecal incontinence severity between patients with IPAA and control group. Wexner scores remained comparable between the two groups at baseline (*p* = 0.9), 6 months (*p* = 0.2), 1 year (*p* = 0.08), 2 years (*p* = 0.17), 3 years (*p* = 0.34), 4 years (*p* = 0.67), and 5 years (*p* = 0.84)

**Fig. 7:**
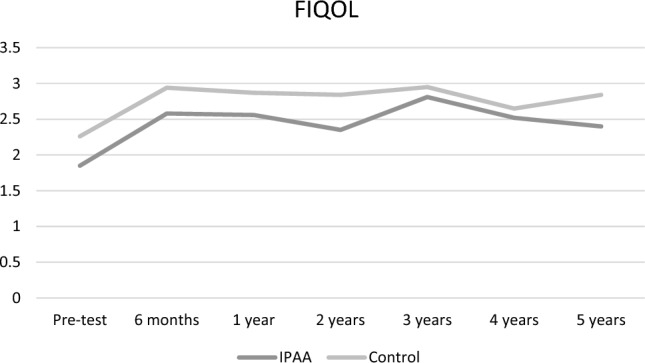
Comparison of quality of life between patients with IPAA and control group. FIQOL scores remained comparable between the two groups at baseline (*p* = 0.12), 6 months (*p* = 0.16), 1 year (*p* = 0.19), 2 years (*p* = 0.08), 3 years (*p* = 0.59), 4 years (*p* = 0.94), and 5 years (*p* = 0.37)

The comparison of both groups over time did not show any significant difference in Wexner scores (Fig. 6) and quality of life (Fig. 7).

## Discussion

This study aimed to assess the results of SNM in patients with bad functional outcome following TPC-IPAA for UC. We found that functional outcome was improved in these patients since there was a decrease of stool frequency, leak episodes, fecal incontinence severity, and a correlative improvement of quality of life. The test was successful in 85% of the patients, which is close to the rates reported in patients with fecal incontinence [9, 10]. After a 5-year follow-up, SNM was still in use in half of the patients, with benefits maintaining over time.

Our results are consistent with those of previously published series. Seifarth et al. reported the results of SNM in 23 patients with IPAA suffering from increased stool frequency or fecal incontinence [8]. The test was unsuccessful in seven (30%) patients. In addition, 16 patients were given definitive implantation. A decrease of fecal incontinence severity—St. Mark’s score decreased from 19 to 4—and stool frequency were noticed. However, there was a high number of missing data in this retrospective study, as a comparison from baseline data was only available for eight patients concerning St. Mark’s scores and for only three patients regarding stool frequency. A systematic review reporting the results of ten patients drew the same conclusions, as fecal incontinence episodes, daily stool frequency, and fecal incontinence severity were improved [7]. However, none of the previously published studies reported an improvement in quality of life. In our series, patients’ quality of life was assessed using the specific FIQOL questionnaire [12]. We could emphasize that quality of life was strongly improved by SNM and that the benefits were also maintained at the end of the follow-up.

Concerning safety, none of the patients experienced any complication following electrode disposal or generator implantation. In a previous pilot study assessing the safety and efficacy of SNM in patients with active UC resistant to medical treatment, we did not report any complication related to the device implantation [13]. The tolerance of the therapy was excellent in all patients and no exacerbation of colitis was noticed. Conversely, a response or a remission was highlighted in half of the patients. Clinical effectiveness of SNM in patients with UC has been confirmed in a comparative Chinese study where 23 patients were submitted to SNM or sham SNM [14]. A clinical response was achieved in 73% of the patients in the SNM group as compared with 27% in the sham group. These data raise the possibility of antiinflammatory effects of SNM in UC and should lead to the consideration of SNM, even in patients with bad functional outcome related to refractory pouchitis.

Currently, SNM mechanisms of action remain not fully understood. Indeed, continence physiology results from a complex interaction between several mechanisms involving not only anal sphincter but also intestinal transit, rectal compliance, and sensitivity along with pelvic reflexes. The effectiveness of SNM, even in patients with sphincter disruption, rectal resection, or TPC-IPAA, leads to the assumption that SNM could exert central effects [7, 8, 10, 15]. In a rodent model of colitis, Tu et al. investigated the mechanisms of action of SNM by performing sacral and vagal nerve sections at different levels before initiating SNM therapy [11]. It has been found that positive effects of SNM were only found in rats with intact sacral afferent and vagus efferent nerves. They concluded that SNM effects were mediated by a pathway involving spinal nerves, brain stem, vagus nerve, and the colon. 

Functional outcome following TPC-IPAA can be impaired by increased stool frequency, fecal urgencies, or fecal incontinence and can be difficult to predict. In a recent meta-analysis, Stephens et al. reported that the results of preoperative anorectal manometry could be predictive of postoperative function following IPAA [16]. Particularly, mean resting pressure was correlated to the Wexner score. This can be of particular interest to guide the shared decision-making before performing IPAA. In our practice, we are used to indicating anorectal manometry within the workup of patients without IPAA suffering from fecal incontinence before considering SNM. However, interpretation of the values must be done cautiously in patients with IPAA, as normal physiology is completely modified by the surgical resection and reconstruction. It can be considered that anorectal manometry results will not influence therapeutic strategy except if an asynchronism is diagnosed. Thus, we do not perform systematic anorectal manometry in the pretreatment workup of patients with IPAA suffering from fecal incontinence.

Our encouraging results are of particular interest because SNM is generally proposed before considering definitive stoma formation. Indeed, therapeutic options in case of bad functional outcome in patients with IPAA are generally limited to lifestyle modifications, antidiarrheal medications, and retraining. In the retrospective study of Seifarth, among the seven patients who failed for SNM, three underwent pouch excision and two received ileostomy [8]. In our experience, among the two patients with test failure, one remained stoma-free despite a bad function. All the patients with secondary SNM interruption were given definitive ileostomy. However, it is important to notice that among the five patients in whom SNM was discontinued, only two were due to loss of efficacy, as other causes were related to rectal cancer recurrence, anoperineal fistula, or pouch Crohn’s disease. In current literature, we can found a 5–10% rate of pouch failure related to chronic complications (anastomotic stricture, septic complications, and refractory pouchitis) or impaired functional outcome [17]. SNM could improve the rate of pouch preservation in that way.

The comparison done with routinely treated patients with FI in our study revealed that the benefits of SNM are equal in terms of incontinence severity and improvement in quality of life. The multicenter study of the French fecal registry study group reported a significant decrease of leakage episodes and fecal urgencies and an improvement of deferment time and quality of life with a high patient satisfaction [10]. Adverse events were low and dominated by pain and infection, which are generally easy to manage but can also compromise therapy continuation. Another study reported long term benefits of SNM up to 10 years after implantation [18]. All these data support extending the indications of SNM to patients with bad functional outcome following TPC-IPAA.

This study has several limitations. First, the sample is small but logically explained by the fact that SNM is poorly proposed in this indication. However, the data completion is comprehensive, especially as compared with previous studies. Second, the use of a specific questionnaire to assess IPAA-related symptoms would have been more suitable. Recently, the PROPS scientific committee developed a specific score called the Ileoanal Pouch Syndrome Severity Score [19]. It explores a wide range of symptoms (leakage, soiling, urgency, bowel movements, time spent on toilet, stool clustering, perianal discomfort, and nocturnal symptoms) and consequences on patients’ daily life (need to plan activities, diet changes, sleep modifications, job adjustments, behavior consequences, mental impact, pad wearings, and need for medications). Unfortunately, this score was not available at the time of the study.

## Conclusions

SNM is effective in the treatment of patients suffering from functional disorders following IPAA. This treatment provides a significant decrease of leaks and improvement of fecal incontinence severity and quality of life. The effectiveness seems comparable to these of patients routinely treated by SNM for fecal incontinence. SMN indications could thus be extended to patients with IPAA presenting bad functional outcome. 

## Data Availability

No datasets were generated or analyzed during the current study.
